# Single-Cell Cytokine Gene Expression in Peripheral Blood Cells Correlates with Latent Tuberculosis Status

**DOI:** 10.1371/journal.pone.0144904

**Published:** 2015-12-14

**Authors:** Pooja Vir, Riccardo Arrigucci, Karim Lakehal, Amy L. Davidow, Richard Pine, Sanjay Tyagi, Yuri Bushkin, Alfred Lardizabal, Maria Laura Gennaro

**Affiliations:** 1 Public Health Research Institute, New Jersey Medical School, Rutgers, The State University of New Jersey, Newark, New Jersey, United States of America; 2 Department of Biostatistics, School of Public Health, New Jersey Medical School, Rutgers, The State University of New Jersey, Newark, New Jersey, United States of America; 3 Global Tuberculosis Institute, New Jersey Medical School, Rutgers, The State University of New Jersey, Newark, New Jersey, United States of America; University of Cape Town, SOUTH AFRICA

## Abstract

RNA flow cytometry (FISH-Flow) achieves high-throughput measurement of single-cell gene expression by combining in-situ nucleic acid hybridization with flow cytometry. We tested whether antigen-specific T-cell responses detected by FISH-Flow correlated with latent tuberculosis infection (LTBI), a condition affecting one-third of the world population. Peripheral-blood mononuclear cells from donors, identified as positive or negative for LTBI by current medical practice, were stimulated ex vivo with mycobacterial antigen. IFNG and IL2 mRNA production was assayed by FISH-Flow. Concurrently, immunophenotypes of the cytokine mRNA-positive cells were characterized by conventional, antibody-based staining of cell-surface markers. An association was found between donor LTBI status and antigen-specific induction of IFNG and IL2 transcripts. Induction of these cytokine genes, which was detected by FISH-Flow in a quarter the time required to see release of the corresponding proteins by ELISA, occurred primarily in activated CD4+ T cells via T-cell receptor engagement. Moreover, NK cells contributed to IFNG gene induction. These results show that antigen-driven induction of T-cell cytokine mRNA is a measurable single-cell parameter of the host responses associated with latent tuberculosis. FISH-Flow read-outs contribute a multi-scale dimension to the immunophenotyping afforded by antibody-based flow cytometry. Multi-scale, single-cell analyses may satisfy the need to determine disease stage and therapy response for tuberculosis and other infectious pathologies.

## Introduction

Flow cytometry allows simultaneous, multiparametric analysis of physico-chemical and functional characteristics of hundreds of thousands of individual cells [1]. Conventional flow cytometry typically analyzes protein abundance by staining protein targets with fluorescently labeled antibodies. In addition, a new technique—RNA flow cytometry—has been developed to detect expression of specific transcripts labeled with fluorescent, complementary nucleic acid probes [2–4]. RNA flow cytometry is effective even when transcripts are present at only a few copies (<10) per cell [3, 4]. We and others have described the versatile use of RNA flow cytometry for analysis of diverse cellular responses and reported on the technical and biological advantages over conventional methods [2–4]. In particular, these initial reports provided proof-of-principle evidence that the method can detect specific T cell responses to stimulation with bacterial and viral antigens [3, 4]. We now turn to determine whether RNA flow cytometry is applicable to the identification of a pathological condition in a patient population.

In the present work we applied RNA flow cytometry, which we call FISH-Flow, to the detection of latent (asymptomatic) infection with *Mycobacterium tuberculosis* (LTBI). This condition, which affects one-third of the world population [5], is suitable for evaluating FISH-Flow because it is diagnosed by assessing antigen-specific T cell responses in vivo, using the tuberculin skin test [6], or ex vivo, by detecting the release of IFN-γ from antigen-specific T cells using ELISA or ELISPOT [7]. We conducted a case-control study involving >60 blood donors to determine whether antigen-specific induction of Th1 cytokine transcripts detected by FISH-Flow correlates with known LTBI donor status. We also examined the nature of the FISH-Flow mRNA signal in LTBI+ donors by characterizing their mRNA-producing T cell subsets and assessing dependence of the signal on T-cell-receptor signaling. The data demonstrate that cytokine mRNA induction in activated T cells constitutes a novel, single-cell parameter of the host responses associated with latent tuberculosis. Combining RNA flow cytometry with conventional, antibody-based flow cytometry can be used for multi-scale immunophenotyping in immunological studies and in clinical research.

## Materials and Methods

### Ethics Statement

Adult (> 18 yrs. of age) subjects were screened and enrolled in this study after written informed consent was obtained at the Lattimore Practice Clinic of the New Jersey Medical School in Newark, NJ and the Middlesex County Chest Clinic. This study was conducted under a protocol approved by the Rutgers University Health Sciences Institutional Review Board.

### Study population and method of enrollment

Adult (> 18 yrs. of age) subjects were sequentially screened and enrolled in this study after written informed consent was obtained at the Lattimore Practice Clinic of the New Jersey Medical School in Newark, NJ and the Middlesex County Chest Clinic. This study was conducted under a protocol approved by the Rutgers University Health Sciences Institutional Review Board. Documentation of LTBI status was a requirement for enrollment. Enrolled individuals were included in the LTBI+ or LTBI- groups based on results obtained during clinical care with either the QuantiFERON-TB interferon gamma release assay (Cellestis, Valencia, CA) or the tuberculin skin test (TST), in accord with current medical practice (http://www.cdc.gov/immigrantrefugeehealth/exams/ti/panel/tuberculosis-panel-technical-instructions.html). Subjects in both groups were asymptomatic and had a negative chest x-ray. Demographics (S1 Table) and medical and clinical information were reviewed by a clinician before entry into the database.

### PBMC collection and cell culture

Within two hours after blood collection, peripheral blood mononuclear cells (PBMCs) were isolated by Ficoll-Paque Plus (GE Healthcare, Waukesha, WI) density-gradient centrifugation and stored in liquid nitrogen. Frozen PBMCs were thawed, washed and cultured in RPMI 1640 supplemented with 2mM L-Glutamine, 1 × penicillin streptomycin solution (Corning Cellgro, Manassas, VA, USA), and 10% heat inactivated Fetal Bovine Serum (Seradigm Providence, UT). All incubation steps were performed at 37°C in a 5% CO_2_ humidified atmosphere.

### PBMC stimulation and cell-surface marker staining

PBMCs (1–2 × 10^7^) were resuspended in 1 ml of culture medium containing 0.1 μg/ml of antibodies against CD28/CD49d (BD Biosciences, San Jose, CA), incubated on ice for 30 min, washed, resuspended in culture medium, and seeded in 24-well tissue culture plates at a cell concentration of 2 × 10^6^ cells/well in 1 ml of culture medium. Stimulation with tuberculin purified protein derivative (Staten Serum Institute, Copenhagen, Denmark) was performed at the indicated concentrations and incubation times. When indicated, PBMCs were stimulated with 1 μg/ml *Staphylococcus aureus* enterotoxin B (SEB) (EMD-Millipore Calbiochem, Billerica, MA) or with a mixture of phorbol 12-myristate 13-acetate (PMA) (Sigma-Aldrich, St Louis, MO) and ionomycin calcium salt (Enzo Life Sciences, Farmingdale, NY) at a final concentration of 25 ng/ml and 0.5 μM, respectively.

After 6 hr of PPD stimulation (unless stated otherwise), PBMCs were transferred to microcentrifuge tubes and washed in PBS-for-FISH (0.2 mg/ml RNAse-free BSA in 1 × PBS [Ambion, Austin, TX], pH 7.4). Cells were resuspended in 50 μl of PBS-for-FISH containing 5 μl of Fc receptor blocking solution, FcX (BioLegend, San Diego, CA), and incubated at room temperature for 5 min. After incubation, 50 μl PBS-for-FISH containing surface marker antibody was added to each tube, and samples were incubated at 4°C for 30 min. The following antibodies were used: CD3 FITC (clone HIT3a), CD3 BUV395 (clone UCHT1), CD4 BV421 (clone RPA-T4), CD4 PE-CF594 (clone RPA-T4), CD8 PE (clone RPA-T8), TCR ϒδ FITC (clone B1), CD56 PE-CF594 (clone B159), CD69 FITC (clone FN50), CD154 BV421 (clone TRAP1). For isotype control staining of CD3 surface marker, FITC-conjugated mouse IgG2a κ antibody was used. The antibody against CD154 was added in co-culture as described by Chattopadhyay et al [8] but without addition of monensin, except when indicated. All antibodies were purchased from BD Biosciences (San Jose, CA).

### Evaluation of T cell activation-specific response in PPD-stimulated samples

PBMCs were resuspended at a cell density of 2 × 10^6^ cells/ml in culture medium containing 0.1 μg/ml of antibodies against CD28/CD49d and stimulated in 24-well tissue culture plate with 10 μg/ml PPD or with immobilized anti human CD3 antibody (1 μg/ml, clone UCHT1), for the indicated times. Recombinant IL-12 (p70) and IL-18 (InvivoGen, San Diego, CA) were used at a final concentration of 10 ng/ml; Cyclosporin A (Sigma, St Louis, MO), anti-IL-12 antibody, and isotype IgG1 κ control antibody were used at 1 μg/ml. Antibodies were purchased from BD Biosciences (San Jose, CA).

### FISH probes

Probe sets for each target mRNA were designed using a dedicated program available at http://www.singlemoleculefish.com and synthetized with a 3’-amino modification by Biosearch Technologies (Novato, CA). The negative control gene was the d2EGFP version of green fuorescent protein [9]. For each gene, a pool of 40–48 oligonucleotides at equal concentrations was prepared, conjugated with 1 mg of Cy5 Bis-Reactive dye (GE Healthcare Life Science, Waukesha, WI), and purified by HPLC as described [9].

### Nucleic acid hybridization (FISH)

After incubation with antibody in the protocols described above, cells were washed in PBS-for-FISH, resuspended in 4% paraformaldehyde (Electron Microscopy Science, Hatfield, PA) in 1 × PBS, and incubated at 4°C overnight or at room temperature for 30 min. Cells were washed in PBS-for-FISH and permeabilized for 30 min at room temperature with 70% Ethanol or with 0.2% Tween 20 (Sigma, St Louis, MO) in 1 × PBS, as indicated. Permeabilized cells were washed in Hybridization Wash Buffer (HWB) (10% formamide, 2 × SSC, 0.2 mg/ml RNAse free BSA, prepared in RNAse free H_2_O), resuspended in 50 μl of Hybridization Buffer (10% dextran sulfate, 10% formamide, 2 × SSC, 0.2 mg/ml RNAse free BSA, 1 mg/ml *E*. *coli* tRNA, in RNAse free H_2_O) containing fluorophore-conjugated mRNA probes (5 ng/sample) and incubated at 37°C overnight. Cells were then washed twice in HWB and resuspended in the same buffer for flow cytometry. All reagents were obtained from Ambion, Austin, TX, except for dextran sulfate and *E*. *coli* tRNA (Sigma, St. Louis, MO).

### Intracellular cytokine staining

After antibody staining of cell surface markers, cells were washed in PBS-for-FISH, resuspended in 4% paraformaldehyde in 1 × PBS, incubated for 30 min at 4°C, washed in PBS-for-FISH, and stored at 4°C overnight. On the following day, cells were resuspended in 500 μl of 1 X Perm/Wash buffer (BD Biosciences, San Diego, CA) and incubated for 10 min at room temperature. After centrifugation, cells were resuspended in 100ul 1 x Perm/Wash buffer containing IFN-γ Alexa 647 antibody (clone B27) (BD Biosciences, San Diego, CA) and incubated at 4°C for 30 min. Cells were washed twice in 1 x Perm/Wash buffer and resuspended in 1% paraformaldehyde for analysis by flow cytometry.

### Quantification of IFN-γ and IL-2 in culture supernatants

Supernatants from PPD-stimulated PBMC cultures were collected by centrifugation and stored frozen at -80°C. Levels of IL-2 and IFN-γ in culture supernatants were assayed using commercial ELISA kits (OptEIA^™^ Human IFN-γ and IL-2 ELISA kits II [BD Biosciences, San Jose, CA]), according to manufacturer’s instructions.

### Flow cytometry

**Fig 1:**
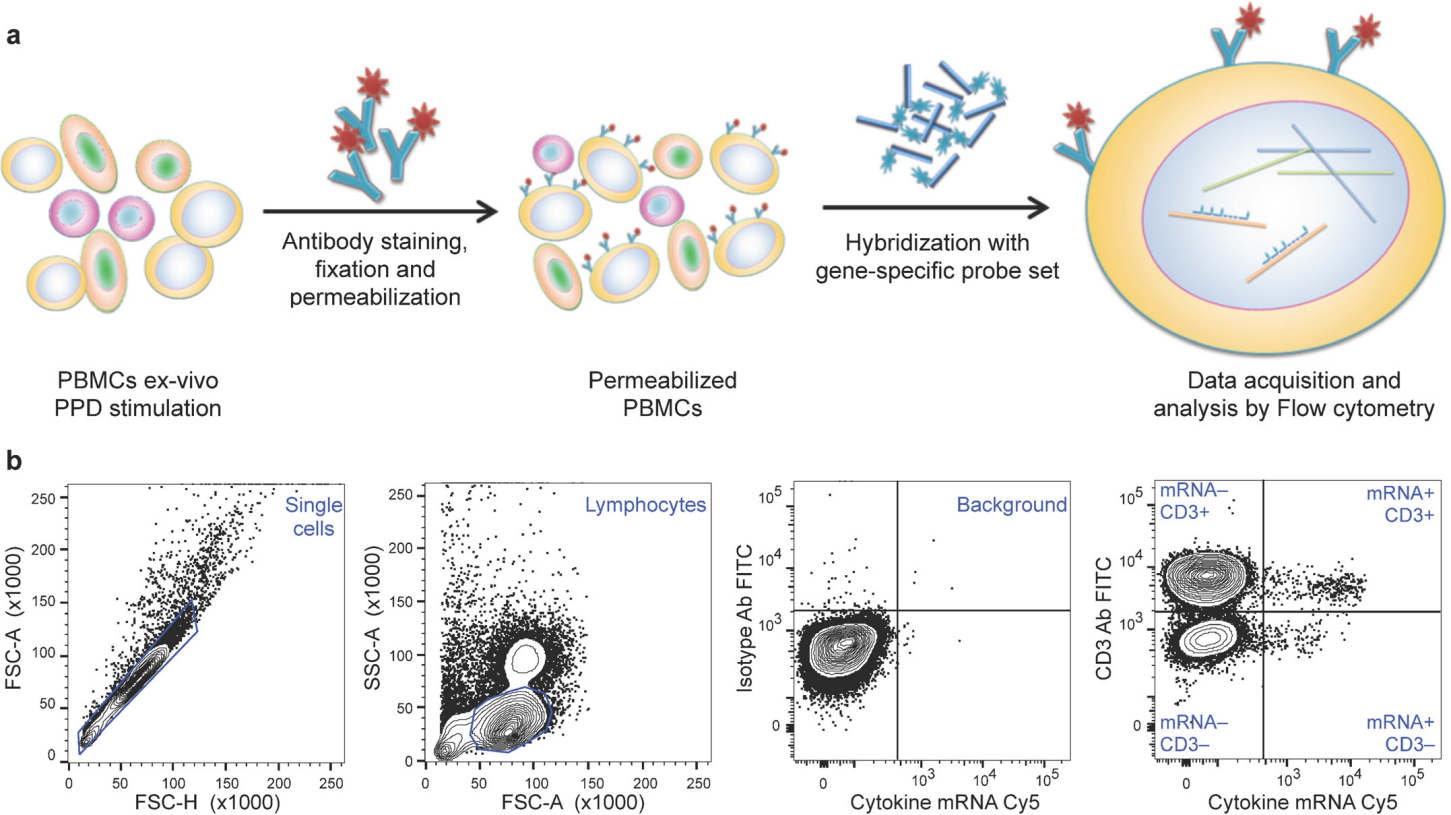
Schematic representation of the dual-color FISH-Flow assay. **(a)** Human PBMCs were left unstimulated or stimulated with 10 μg/ml PPD for 6 hr, in the presence of costimulatory monoclonal antibodies (αCD28 and αCD49d, each at 0.1 μg/ml). The cells were then stained with FITC-labeled αCD3 antibody, fixed, permeabilized, and hybridized overnight with Cy5-labeled, gene-specific nucleic acid probes. After hybridization, 100,000 events were acquired on a BD LSRII flow cytometer and analyzed. **(b)** Gating strategy: Single cells (inside the diagonal gate) were selected in the forward scatter-height (FSC-H) versus forward scatter-area (FSC-A) plot. Lymphocytes were then identified in a FSC-A versus side scatter-area (SSC-A) plot. Unstimulated cells stained with FITC-labeled isotype-control antibody and Cy5-labeled nucleic acid probe were gated in a bivariate plot to identify background. These gates were applied to unstimulated and stimulated cells stained with FITC-labeled αCD3 antibody and Cy5-labeled nucleic acid probe to obtain the frequencies of events in each quadrant. The events in the upper right quadrant (double-positive cells) were used for subsequent analysis.

Data were acquired on an LSRII flow cytometer (BD Biosciences, San Jose, CA) and analyzed with FACSDiva (BD Biosciences, San Jose, CA) or FlowJo X.0.7 (TreeStar, Ashland, OR) software. Data were acquired using the bichromatic gating strategy described in Fig 1b. For each experimental condition, 100,000–200,000 total events were acquired. In multicolor experiments, spectral compensation values for fluorochrome-labeled antibodies were calculated using polystyrene compensation beads (BD Biosciences, San Jose, CA), according to the manufacturer's protocols. For mRNA probes, PMA-Ionomycin-stimulated lymphocytes unstained and stained with Cy5-labeled IFNG probes were used as compensation controls. Data were acquired using a 355 nm laser with a 379/28 BP filter for the detection of BUV395; a 405-nm laser with a 450/40 BP filter for the detection of BV421; a 488-nm laser with a 530/30 BP 505 LP filter for the detection of FITC, a 575/26 BP 550 LP filter for the detection of PE, a 610/20 BP 600 LP filter for the detection of PE-CF594; and a 640-nm laser with a 660/20 BP filter for the detection of Cy5 probes.

### Statistical analysis of flow cytometry data

For each target gene, Poisson regression models of the proportion of CD3+ cells that were mRNA+ were estimated using the SAS PROC GENMOD module (SAS Version 9.3). Using a log link function, counts of mRNA+CD3+ cells were regressed on LTBI status (positive vs. negative), stimulation type (unstimulated vs. PPD-stimulated), and an interaction term (LTBI status x stimulation). The total number of CD3+ cells was entered into the model as an offset variable. Counts were assumed to follow a Poisson distribution with over-dispersion, i.e., the variance of the counts was allowed to be larger than expected under the Poisson assumption. The over dispersion parameter was estimated from Pearson’s chi-square statistic divided by the degrees of freedom [10]. The generalized estimating approach was used to adjust for intra-subject correlation of mRNA+CD3+ counts with and without PPD stimulation [11]. Model coefficients were exponentiated to derive ratios of proportions of LTBI+ to LTBI- by stimulation type. Confidence intervals for these ratios of proportions as well as associated p-values were also obtained from these models.

Fluorescence values were analyzed as follows. For each target gene and each stimulation type, the mean of the log-fluorescence values was estimated for each subject. Then, separately for each of the six target gene-stimulation type combinations, a non-parametric test (Mann-Whitney U test) comparing the mean log-fluorescence values of the LTBI positive subjects with LTBI negative subjects was conducted.

## Results

### FISH-Flow detection of antigen-induced cytokine mRNA

We set out to determine whether the antigen-specific T cell responses associated with LTBI can be detected by an immunoassay utilizing FISH-Flow. With this technique, mammalian cells in suspension are fluorescently stained for surface markers using antibody and for mRNA using nucleic acid probes, and analyzed by flow cytometry (Fig 1). We developed a protocol for detecting antigen-induced expression of the cytokine genes IFNG and IL2 in peripheral blood T cells stimulated ex vivo with *M*. *tuberculosis* purified protein derivative (PPD), a standard reagent used for recall responses in tuberculosis [6]. In these assays we used a green fluorescent protein (GFP) gene as a negative control. Pilot experiments showed that a 6-hr stimulation with 10 μg/ml PPD was adequate for cytokine mRNA detection (S1 Fig). This incubation time was much shorter than the 24 hr that was required for maximal detection of the corresponding protein analytes (S2 Fig). This latter result was in accord with a large body of tuberculosis literature where cytokine production and secretion are typically recorded at 24 or 72 hrs (examples are [12–14]).

**Fig 2:**
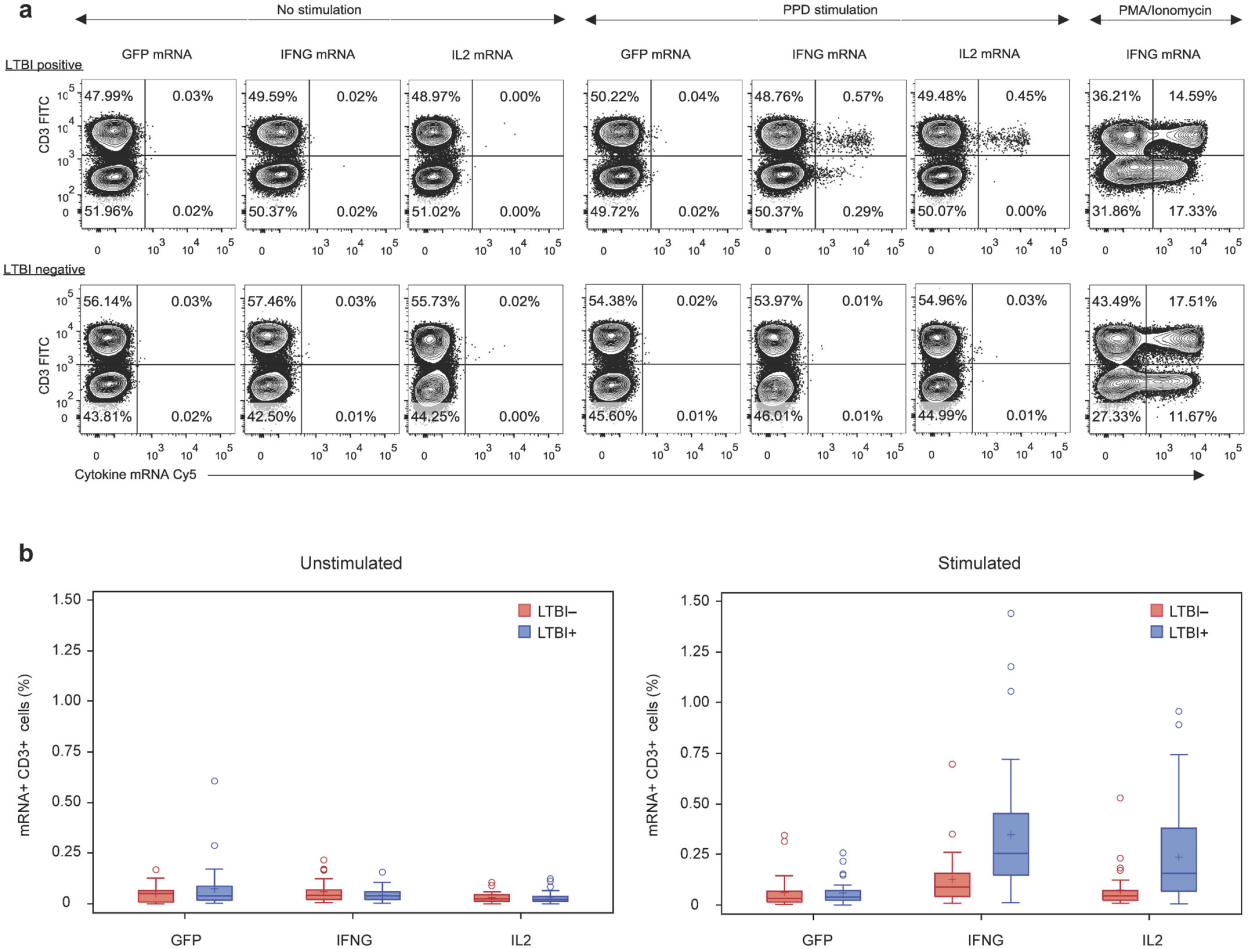
FISH-Flow detection of antigen-specific T cell responses associated with latent tuberculosis infection. PBMCs from 65 LTBI+ and LTBI- donors were cultured +/- PPD stimulation, stained with FITC-labeled αCD3 antibody, hybridized with Cy5-labeled nucleic acid probes specific for IFNG, IL2, and no target control (GFP) and analyzed by flow cytometry, as detailed in the legend to Fig 1. (a) Representative contour plots showing results with cells from an LTBI+ (top row) and an LTBI- (bottom row) donor, unstimulated and PPD-stimulated, as indicated. The rightmost panels in both rows show IFNG induction with PMA/Ionomycin stimulation as positive control. The upper right quadrant of each plot shows the frequencies of mRNA+CD3+ cells. (b) Frequencies of mRNA+CD3+ cells in the 65-donor population, by LTBI and stimulation status. The box plots show lower quartile, median, and upper quartile of the distribution. The lower whisker is the minimum, while the upper limit of the whisker represents the median + 1.5 x the interquartile range (values exceeding the upper limit of the whisker are shown as circles). Additionally, the mean is shown as (+) symbol. To confirm the reproducibility of the assay, selected donors were assayed twice with blood samples drawn > 4 months apart.

We next performed a case-control study on 65 subjects having known LTBI status as determined by standard tuberculosis control practice (see Methods). The study subjects were divided almost equally between LTBI+ and LTBI- individuals (donor characteristics are listed in S1 Table). For each donor, PBMC (+/- PPD stimulation) were fluorescently stained with FITC-labeled anti-CD3 antibodies and with Cy5-labeled nucleic acid probe sets for IFNG, IL2, and GFP mRNA. Representative results are shown in Fig 2a in which an LTBI+ donor exhibited increased T cell expression of IFNG and IL2 mRNA in response to antigen stimulation (>25 fold and >40 fold, respectively, relative to unstimulated samples) (top row). Little or no antigen-induced cytokine gene expression by T cells was observed with an LTBI-negative donor sample (bottom row). No response to antigen stimulation was observed in cells labeled with the control GFP probe, regardless of donor LTBI status (Fig 2a). When we computed the results obtained with all 65 donors, we found that LTBI status was associated with significant differences in the frequencies of antigen-stimulated T cells expressing IFNG and IL2 mRNAs (*p* <0.002) but not GFP mRNA (*p* = 0.58) (Fig 2b and Table 1). In contrast, no significant difference was detected between the two donor classes for IFNG and IL2 gene expression in the absence of antigen stimulation (*p* >0.11) (Fig 2b and Table 1). Furthermore, when we analyzed the data in terms of Cy5-fluorescence intensity of mRNA+CD3+ cells, we also found statistically significant differences with respect to LTBI status for both IFNG and IL2 mRNA induction in T cells following PPD stimulation (*p* = 0.001 and 0.046, respectively), but not in the absence of antigen stimulation (*p* = 0.17 and 0.77, respectively) (S3 Fig). No significant difference was observed for GFP mRNA, regardless of stimulation status (*p* ≥0.41; S3 Fig). Collectively, these data show that the antigen-specific T cell responses detected by FISH-Flow are associated with LTBI status.

**Table 1:** Effect of LTBI status and PPD stimulation on target gene expression.

	LTBI+ vs LTBI- (PPD stimulation)		LTBI+ vs LTBI- (No stimulation)	
Target gene	Ratio of proportions [C.I.]	p-value	Ratio of proportions [C.I.]	p-value
**GFP**	1.2 [0.7, 2.1]	0.58	1.2 [0.7, 2.1]	0.38
**IFNG**	2.5 [1.5, 4.3]	<0.001	0.7 [0.4, 1.1]	0.11
**IL2**	2.9 [1.5, 5.7]	<0.002	1.0 [0.6, 1.5]	0.95

Ratios of proportions of mRNA+CD3+ cells are shown in LTBI+ vs. LTBI- donors by PPD stimulation. Ratios of proportions and associated confidence intervals (C.I.) and p-values were calculated using a Poisson regression model, with a log-link adjusted for the total number of CD3+ cells.

### Lymphocyte subset analysis of antigen-specific IFNG- and IL2-expressing cells in LTBI+ donors

**Fig 3:**
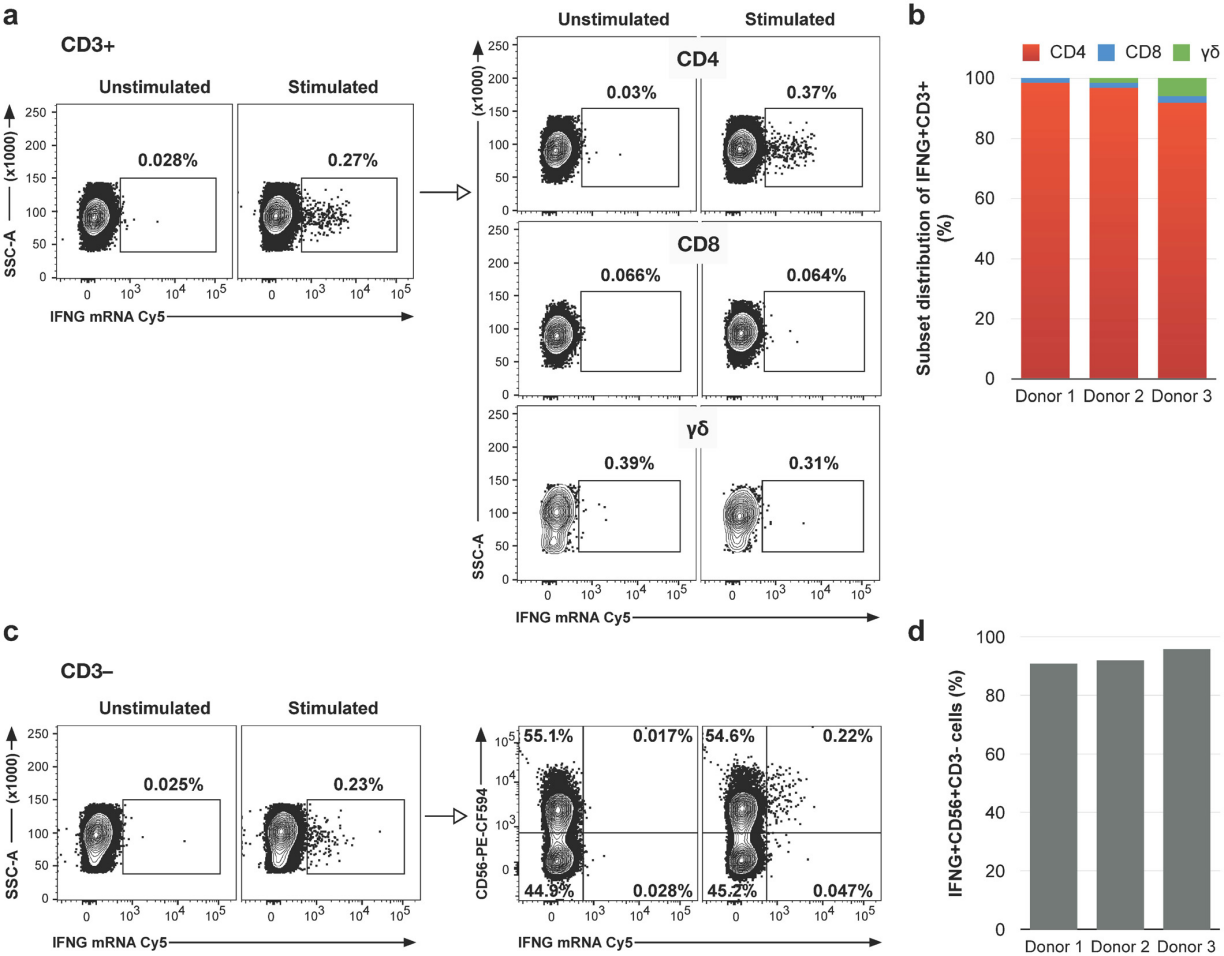
Analysis of IFNG expression in CD3+ and CD3- cell subsets. PBMC from LTBI+ donors were stimulated with PPD or left unstimulated, stained with antibodies against surface markers as indicated, probed with Cy5-labeled IFNG probes, and analyzed by flow cytometry. Gates were set based on unstimulated samples and Fluorescence Minus One (FMO) controls. The scatter plots show data from one donor and the bar graphs from three donors. In each panel, frequencies were calculated relative to the total number of cells in the panel. (a) Frequencies of IFNG+CD3+ cells (left panel) and IFNG+ cells in the CD4+, CD8+ and γδ T cell subsets (right panel). (b) Frequency of CD4+, CD8+, and γδ subsets in IFNG+CD3+ cells. (c) Frequencies of IFNG+CD3- cells (left panel) and IFNG+CD56+ cells (right panel, upper right quadrants). (d) Frequency of CD56 in IFNG+CD3- cells.

To characterize the FISH-Flow signal, we next examined the lymphocyte subsets expressing IFNG and IL2 mRNA following PPD stimulation in LTBI+ donors. This analysis requires multi-color staining of lymphocyte surface markers, which is often associated with use of tandem-dye-conjugated antibodies. Some of these conjugates are incompatible with the ethanol-based permeabilization buffers utilized in previous RNA flow cytometry protocols (for example, [3, 4]). Consequently, we first compared cell permeabilization using ethanol with permeabilization using Tween-20, a non-ionic detergent that is compatible with most dyes used in flow cytometry. The two conditions gave similar frequency and fluorescence intensity for cytokine mRNA+ cells (S4 Fig), indicating that the detergent-based permeabilization is fully compatible with FISH-Flow. Using the Tween-modified protocol, we characterized the T cell subsets associated with the CD3+ response observed in LTBI+ donors. When we stained PBMC with antibodies against CD3, CD4, CD8, and TCR γδ, we found that the vast majority (>90%) of cells inducing IFNG mRNA were CD4+ T cells (Fig 3a and 3b). Similar results were observed for IL2 mRNA induction (S5 Fig).

We also observed that ~30% of LTBI+ donors showed induction of IFNG (but not IL2) mRNA in cells negative for CD3 (for example, Fig 2a top row). To further characterize the CD3-negative cells, we stained LTBI+ donor cells with CD56, a marker of natural killer (NK) cells [15]. We found that >90% of IFNG+CD3-negative cells were positive for CD56 (Fig 3c and 3d). The observed antigen-dependence of IFNG mRNA induction in NK cells strongly suggests that NK cell activation is dependent on the antigen-specific CD4+ T cells [16].

We conclude that CD4+ T cells are the main subset producing IFNG and IL2 mRNA, while NK cells constitute the predominant IFNG+CD3-negative cell population. Moreover, the results show that the FISH-Flow signal is strong enough to be detected concurrently with multiple cell types.

### Mechanism of the antigen-driven cytokine gene induction detected by FISH-Flow

**Fig 4:**
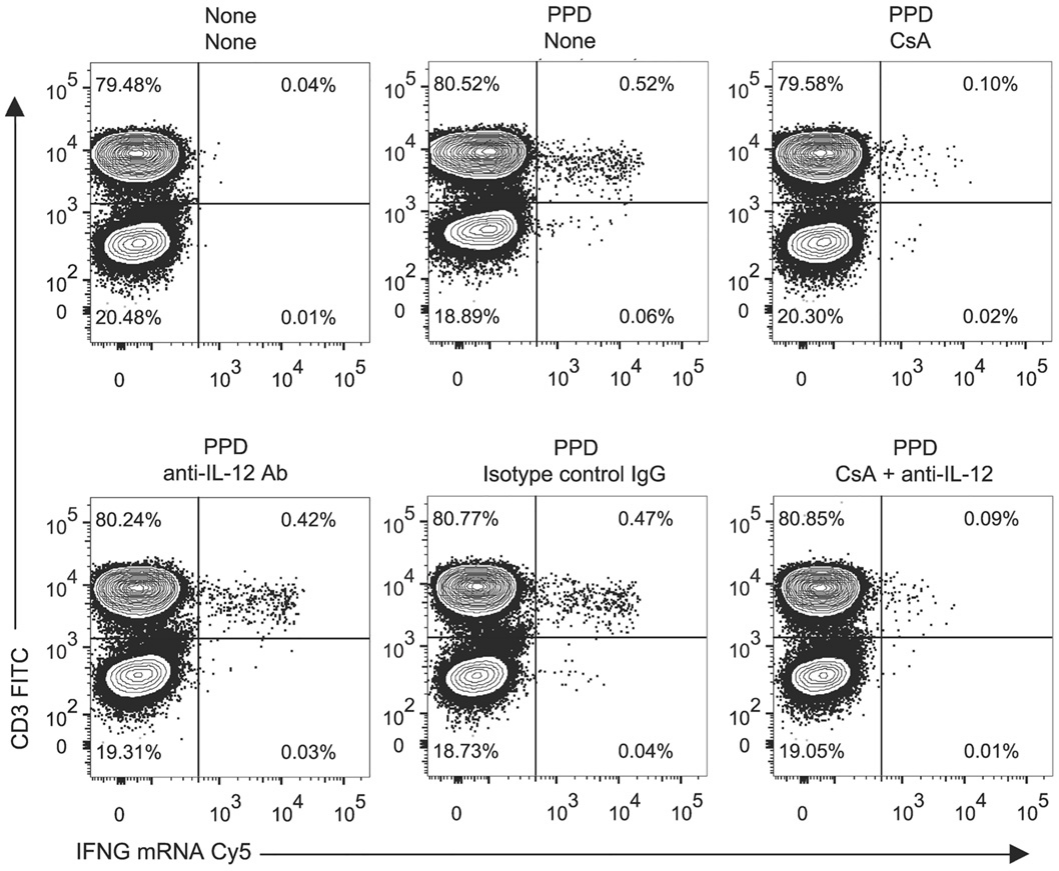
Analysis of the mechanism of PPD-induced IFNG expression in T cells. PBMCs from an LTBI+ donor were treated with CsA, αIL-12, or isotype control antibody for 1 hr and then stimulated with PPD for 6 hr. The frequency of IFNG+CD3+ cells is shown in the upper right quadrant of each bichromatic contour plot. The title above each plot indicates the conditions used for stimulation (top line) and pretreatment (bottom line).

Production of IFN-γ by CD4+ T cells can occur via direct engagement of the T-cell receptor, or it can result from the paracrine action of IL-12 and IL-18 [17]. To further characterize the nature of the FISH-Flow signal, we set out to determine which mechanism was responsible for the cytokine gene induction detected in peripheral blood T cells under the experimental conditions used (6-hr stimulation with PPD). Prior to PPD stimulation, we treated LTBI+ donor cells with an inhibitor of T cell activation, such as cyclosporine [18], or with neutralizing anti-IL-12 antibody (assay conditions were established in preliminary experiments using PBMCs stimulated with anti-CD3 antibody or with IL-12 and IL-18; S6a Fig). Pretreatment of LTBI+ donor cells with cyclosporine reduced expression of IFNG mRNA (75–80%) and IL2 mRNA (>90%) (Fig 4 and S6b Fig). In contrast, no effect was observed when the cells were pretreated with neutralizing anti-IL-12 antibody (Fig 4). Moreover, concurrent pretreatment with cyclosporine and anti-IL-12 antibody afforded no additional inhibition relative to cyclosporine alone (Fig 4). Thus, the antigen-driven cytokine mRNA induction in T cells detected by FISH-Flow after 6-hr PPD stimulation is a response to T-cell receptor signaling rather than to the cytokine environment generated by non-T cells.

### Expression of surface markers of T cell activation in LTBI+ donors

**Fig 5:**
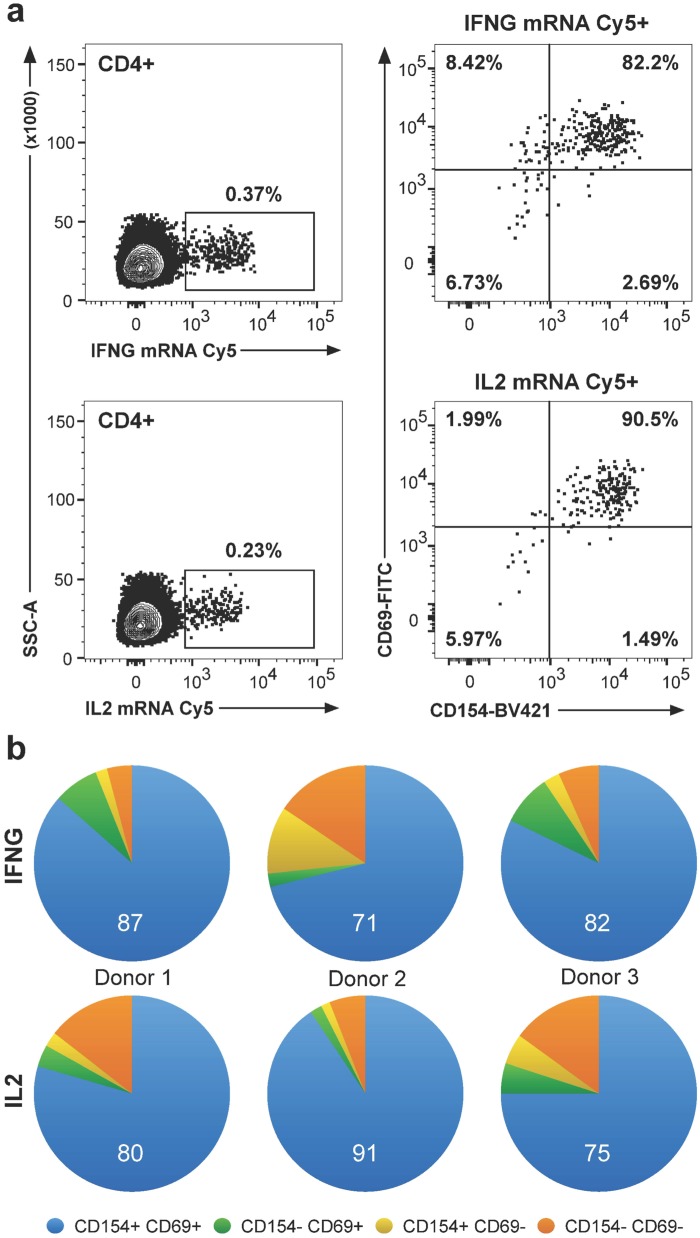
Expression of CD69 and CD154 activation markers in CD4+ T cells. PPD-stimulated PBMC from LTBI+ donors were co-cultured with αCD154 antibody, stained for CD69, CD4, and CD3, probed with Cy5-labeled IFNG and IL2 nucleic acid probes, and analyzed by flow cytometry. Gates were set based on unstimulated negative controls, SEB-stimulated positive controls, and FMO controls. The scatter plots show data from one donor and the pie charts from three donors. **(a)** Frequencies of cytokine mRNA+CD4+ T cells (left panels) and CD69 and CD154 expression in mRNA+CD4+ T cells (right panels). The upper right quadrant shows mRNA+CD69+CD154+ cells. Cytokine: IFNG (top row) and IL2 (bottom row). **(b)** Frequencies of all combinations of CD69 and CD154 expression in IFNG+ (top row) and IL2+ (bottom row) CD4+ T cells, one donor per column.

We next characterized the expression of surface markers of T cell activation in the CD4+ T cells found to be cytokine-gene positive in the FISH-Flow assay. We selected CD69, an early marker expressed by activated T cells [19, 20], and CD154 (CD40L), which is largely restricted to CD4+ T cells that produce cytokines in response to antigen [8, 21]. Due to the transient expression of CD154, detection involves including the anti-CD154 antibody in the culture for the entire duration of antigen stimulation [22]. We did not add the protein-transport inhibitor monensin to the culture, since CD4+ T cells, activated with staphylococcal enterotoxin B in preliminary control experiments showed greater frequency (2.5 fold) of CD154+ cells in the absence than in the presence of inhibitor (S7 Fig). We found that the vast majority (>80%) of antigen-specific, mRNA+CD4+ T cells expressed both CD69 and CD154 activation markers (Fig 5), indicating that the FISH-Flow signal is associated with activated CD4+ T cells.

### Comparison between FISH-Flow and intracellular staining

**Fig 6:**
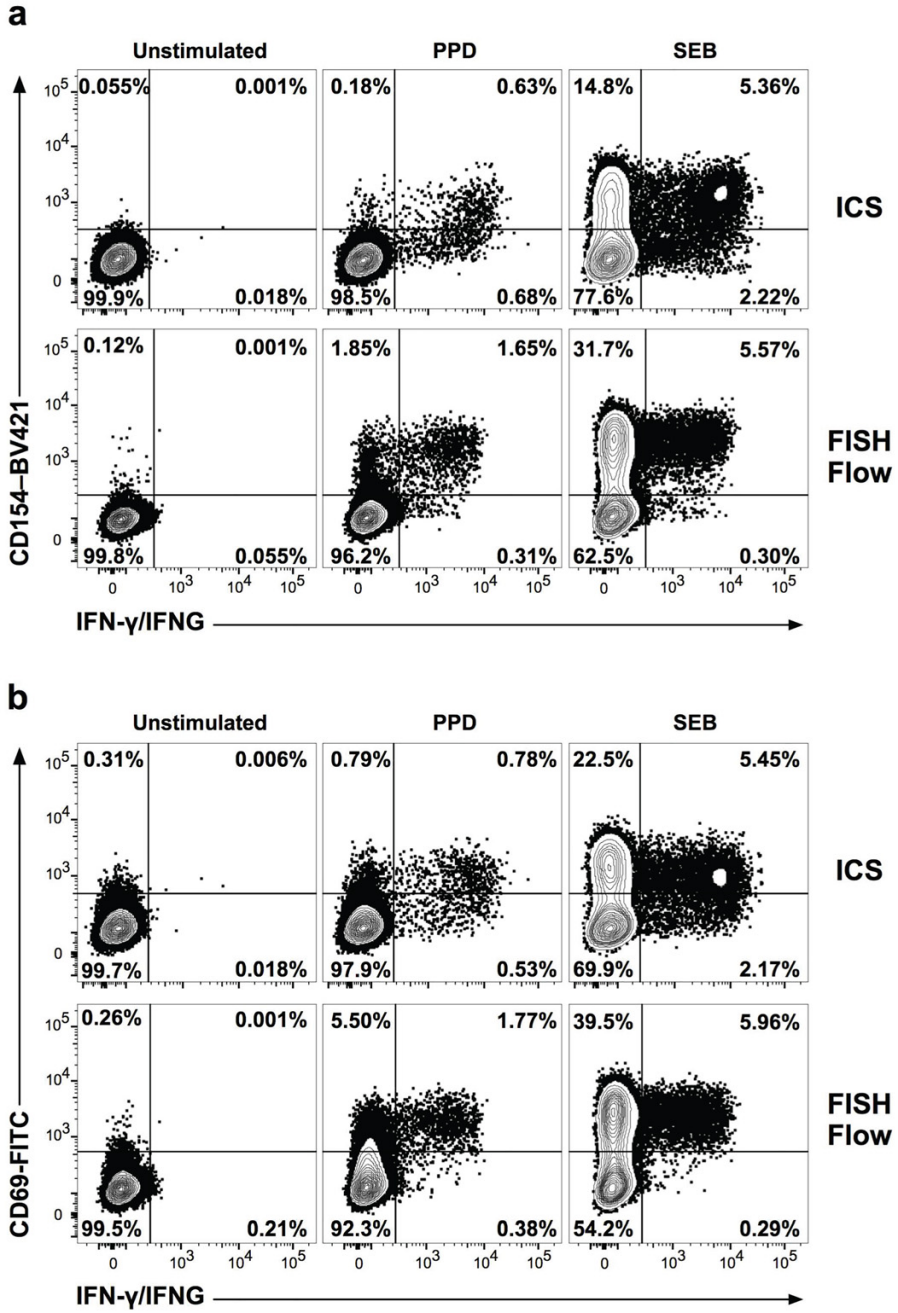
Comparison of FISH-Flow and intracellular cytokine staining (ICS) for detection of antigen-specific T cell responses. PBMC from an LTBI+ donor were stimulated with PPD, SEB, or left unstimulated. All cells were co-cultured with αCD154 antibody, stained for CD69, CD4, and CD3, probed with Cy5-labeled IFNG (FISH-Flow) or Alexa 647 IFN-γ antibody (ICS), and analyzed by flow cytometry. In the ICS assay, samples were incubated in the presence of 2 μM monensin. Gates were based on unstimulated negative controls and FMO controls. **(a)** Frequencies of IFN-γ protein+ (IFN-γ) or IFNG mRNA+ (IFNG) cells among CD4+ T cells expressing the CD154 T cell activation marker. **(b)** Frequencies of IFN-γ protein+ (IFN-γ) or IFNG mRNA+ (IFNG) cells among CD4+ T cells expressing the CD69 T cell activation marker.

Intracellular staining (ICS) for measuring cytokine production involves blocking cytokine release by treatment with protein secretion inhibitors, such as monensin or brefeldin A, followed by staining of the intracellular cytokines with fluorochrome-conjugated antibodies [23]. To compare FISH-Flow and ICS, we analyzed the frequencies of cells expressing IFNG mRNA and IFN-γ protein, respectively, and the expression of CD69 and CD154 T cell activation markers in antigen-stimulated CD4+ T cells from an LTBI+ donor. The frequencies of CD4+ T cells producing IFNG mRNA or IFN-γ protein were similar in the PPD-stimulated samples (not shown). However, the samples processed by FISH-Flow showed a >4-fold increase in the expression level of CD69 and CD154 when compared to ICS samples (Fig 6, middle column, upper left quadrant). Additionally, the overall proportion of activated, cytokine-producing cells detected by FISH-Flow (>80%) was higher than detected by ICS (~50–60%) (Fig 6, middle column, upper right quadrant). Similar results were obtained with cells stimulated with staphylococcal enterotoxin B (SEB) (Fig 6, right column), showing that the effect is not antigen-specific. These results strongly imply that treatment with protein transport inhibitors reduces expression of surface markers of T cell activation, such as CD69 and CD154.

## Discussion

When applied to a case-control study, FISH-Flow distinguished latent *M*. *tuberculosis* infection status of asymptomatic blood donors based on antigen-specific induction of the genes encoding two major Th1 cytokines, IFN-γ and IL-2, in peripheral blood cells. Thus, the ability of FISH-Flow to detect antigen-specific T cell responses, which was previously reported [3, 4], can be applied to identifying a pathological condition. The FISH-Flow signal from induced transcripts was almost exclusively associated with CD4+ T cells, consistent with previous work using conventional methods [24–27]. Cytokine gene induction occurred via T-cell-receptor signaling, a mechanistic aspect that has not been previously reported in studies of antigen-induced T-cell cytokine release in tuberculosis. In some LTBI+ donors, NK cells also contributed to IFNG gene induction; the NK cell contribution depended on T cell activation (Fig 3c, 3d and S6b Fig), as seen in other pathologies [16]. The FISH-Flow method was combined with the analysis of multiple cell-surface markers by conventional flow cytometry by altering the cell permeabilization method to include Tween 20 (S4 Fig). This mild detergent is compatible with all the fluorochromes and tandem dyes used in flow cytometry, unlike the alcohol-based cell permeabilization method described in the previous RNA flow cytometry reports [3, 4]. Thus, FISH-Flow adds a multi-scale dimension to the immunophenotyping afforded by antibody-based, multi-parameter flow cytometry.

Our results support the proposition that RNA flow cytometry offers multiple, technical and biological, advantages over conventional methods for detection of T-cell activation [2–4, 28]. In general, the use of mRNA rather than protein as analyte in flow cytometry greatly broadens the spectrum of cell-marker readouts, since producing nucleic acid probes is more straightforward than obtaining antibodies of suitable quality. Moreover, when applied to cytokine gene detection, as in the present work, RNA flow cytometry overcomes the use of toxic protein-transport inhibitors, which is a staple of intracellular staining. Indeed, we observed that, due to the use of monensin, intracellular-staining-based flow-cytometry underestimated CD154 and CD69 expression by IFNG+ CD4+ T cells (Fig 6 and S7 Fig), a clear indication that transport inhibitors affect the expression of various cell markers. RNA flow cytometry also circumvents the need for prolonged “resting” of PBMC to minimize background signals prior to antigen stimulation of T cells [29], presumably due to more rapid turnover of mRNA relative to protein [3]. Importantly, maximal detection can be obtained at much earlier times for cytokine transcripts than for corresponding protein release (for example, S2 Fig). Indeed, the temporal characteristics of mRNA induction are early enough to distinguish stimulus through T-cell-receptor-dependent activation from response to cytokines released by antigen-presenting cells (Fig 4 and S6 Fig), without confounding feedback paracrine effects.

Due to the combination of high-throughput and multi-parameter properties, flow cytometry is ideally suited for monitoring evolution of cellular subpopulations and phenotypes associated with progression of various infectious and non-infectious pathologies. Indeed, such properties are the basis of the prognostic capabilities of flow cytometry for various pathologies [30–32] including *M*. *tuberculosis* infection outcome [33, 34]. The short half-life of mRNA (relative to protein) (for example, S2 Fig) may provide an additional dimension to conventional flow cytometry by allowing a more dynamic representation of cell state while concurrently maintaining the ability to characterize cell types by conventional, antibody-based staining of cell surface markers. Moreover, parallel measurements of mRNA and protein for the same markers may distinguish between effects, for example caused by infectious agents, on transcriptional and translational regulation in immune cells. The present demonstration that FISH-Flow detects the antigen-specific cytokine production associated with LTBI-positive status constitutes a powerful, first step toward applying FISH-Flow to the study of the complex immunological changes associated with progression of tuberculosis [27, 35] that are not revealed by the current tests for LTBI [36].

In conclusion, the present report opens multiple avenues of future research. These include tuberculosis-specific goals such as (i) determining FISH-Flow cutoffs to assess its accuracy for LTBI diagnosis vis-à-vis current assays; and (ii) utilizing FISH-Flow for the identification of immunophenotypes that may differentiate stable LTBI from progression to active tuberculosis (recent reports provide encouraging results on the usefulness of multi-parameter flow cytometry for this purpose [14, 37]). Moreover, the present work provides further incentive for improving nucleic acid probe design and developing additional, analyte-specific fluorophores to achieve increased signal-to-noise ratios in RNA flow cytometry for research in infectious and non-infectious pathologies. We expect that establishing robust protocol specifications will lead to clinical application of FISH-Flow.

## Supporting Information

S1 Fig Optimization of dose and duration of PPD stimulation in the FISH-Flow assay. PBMCs from an LTBI+ donor were stimulated in culture with PPD at three concentrations and multiple time points, as indicated, and analyzed for IFNG mRNA expression in CD3+ cells by FISH-Flow as described in the legend to Fig 1. Shown are data from one LTBI+ donor out of three tested.
(PDF)

S2 Fig Time course of cytokine mRNA induction and corresponding protein secretion. PBMCs from an LTBI+ donor were stimulated with 10 μg/ml PPD for the indicated times. Stimulated T cells were analyzed for cytokine mRNA by FISH-Flow and cell-free culture supernatants from the same wells were assayed for cytokine protein levels by ELISA. (a) IFN-γ and (b) IL-2. In both panels and for each time point, the right vertical axis (red) shows the frequency of cytokine mRNA expressing CD3+ cells, while the left vertical axis (blue) shows the concentration of secreted protein (in pg/ml).
(PDF)

S3 Fig Cy5 fluorescence intensity of mRNA+CD3+ cells in LTBI+ and LTBI- donors. PBMCs from 33 LTBI+ and 32 LTBI- donors were cultured +/- PPD, stained with FITC-labeled αCD3 antibody, hybridized with Cy5-labeled nucleic acid probes specific for GFP, IFNG and IL2, and analyzed by flow cytometry, as detailed in the legend to Fig 1. The Cy5 fluorescence intensity value of each mRNA+CD3+ cell was extracted using FlowJo software. The graphs show histograms of log-transformed fluorescence data for each gene, unstimulated and PPD stimulated, from LTBI+ and LTBI- donors. Each bin of the histograms comprises interval values of 0.25.
(PDF)

S4 Fig Comparison of permeabilization buffers containing 70% ethanol or 0.2% Tween 20. PBMCs were stimulated for 2 hr with PMA and Ionomycin, fixed in 4% PFA, and permeabilized for 30 min at room temperature with 0.2% Tween 20 (top row) or 70% ethanol (bottom row). After washes, cells were hybridized with Cy5-labeled GFP, IFNG, or IL2 nucleic acid probes, and analyzed by flow cytometry. Cells were gated according to the forward and side light scatter characteristics of viable lymphocytes. Gates were set on the basis of the GFP control probe and unstimulated control samples. Frequencies of cells expressing GFP, IFNG and IL2 mRNA are reported above each gate. Data from a representative experiment are shown. Similar results were obtained with PPD-stimulated PBMC from an LTBI+ donor (data not shown).
(PDF)

S5 Fig Analysis of IL2 expression in CD3+ subsets. PBMC from LTBI+ donors were stimulated with PPD or left unstimulated, stained with antibodies against surface markers as indicated, probed with Cy5-labeled IFNG probes, and analyzed by flow cytometry. Gates were set based on unstimulated samples and Fluorescence Minus One (FMO) controls. The scatter plots show data from one donor and the bar graphs from three donors. In each panel, frequencies were calculated relative to the total number of cells in the panel. (a) Frequencies of IL2+CD3+ cells (left panel) and IL2+ cells in the CD4, CD8 and γδ T cell subsets (right panel). (b) Frequency of CD4, CD8, and γδ subsets in IL2+CD3+ cells.
(PDF)

S6 Fig Analysis of the mechanism of PPD-induced IFNG expression in T cells. (a) PBMCs were stimulated in vitro for 4 hr with either immobilized αCD3 antibody and αCD28/CD49d costimulatory molecules (top row) or with recombinant human IL-12 and IL-18 cytokines (bottom row). Prior to stimulation, cells were subjected to 1-hr treatment at 37°C with CsA, αIL-12 antibody, isotype control antibody, or CsA and αIL-12 antibody together. Gates were established based on unstimulated samples stained with FITC αCD3 antibody and Cy5-labeled IFNG nucleic acid probe. The frequency of IFNG+CD3+ cells is shown in the upper right quadrant of each bichromatic contour plot. (b) PBMCs from an LTBI+ donor were treated with CsA for 1 hr or left untreated, prior to 6 hr PPD stimulation. Stimulated cells were stained with FITC αCD3 antibody, probed with Cy5-labeled nucleic acid probes for IFNG (top panels) or IL2 (bottom panels), and analyzed by flow cytometry.
(PDF)

S7 Fig Surface expression of CD154 activation marker in the presence or absence of monensin. PBMCs were stimulated with SEB for 6hr. αCD154 antibody was added during the stimulation (co-culture method), with or without addition of 2 μM monensin, as indicated. Cells were stained for CD3 and CD4 surface markers, probed with Cy5-labeled GFP and IFNG nucleic acid probes, and analyzed by flow cytometry. Gates were set based on unstimulated samples, GFP control probe, and FMO controls. Frequencies of CD4+ T cells are shown in each quadrant.
(PDF)

S1 Table Demographics of the study population. (DOCX)

S2 Table Reproducibility of the FISH-Flow assay. (DOCX)
