# Supplementary figures and images for: Single-Cell Cytokine Gene Expression in Peripheral Blood Cells Correlates with Latent Tuberculosis Status
Source: PLoS One. 2015 Dec 14;10(12):e0144904. doi: 10.1371/journal.pone.0144904 (PMC4681842; doi:10.1371/journal.pone.0144904)

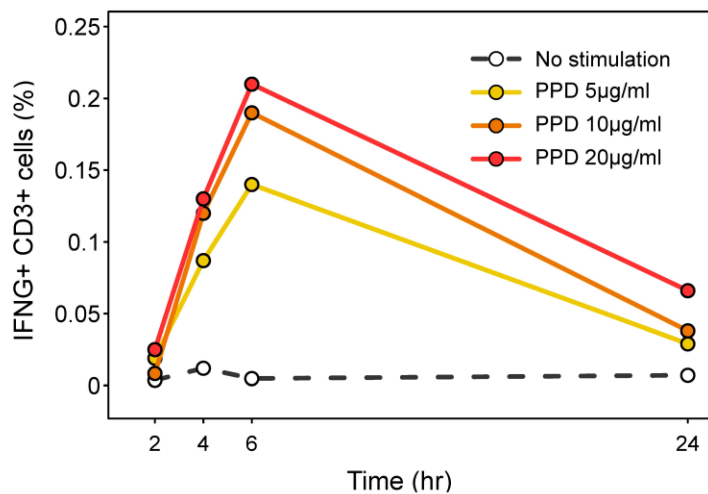

**S1 Fig. Optimization of dose and duration of PPD stimulation in the FISH-Flow assay.**

Supplement: S1 Fig — PBMCs from an LTBI+ donor were stimulated in culture with PPD at three concentrations and multiple time points, as indicated, and analyzed for IFNG mRNA expression in CD3+ cells by FISH-Flow as described in the legend to Fig 1. Shown are data from one LTBI+ donor out of three tested. (PDF) [file pone.0144904.s001.pdf]

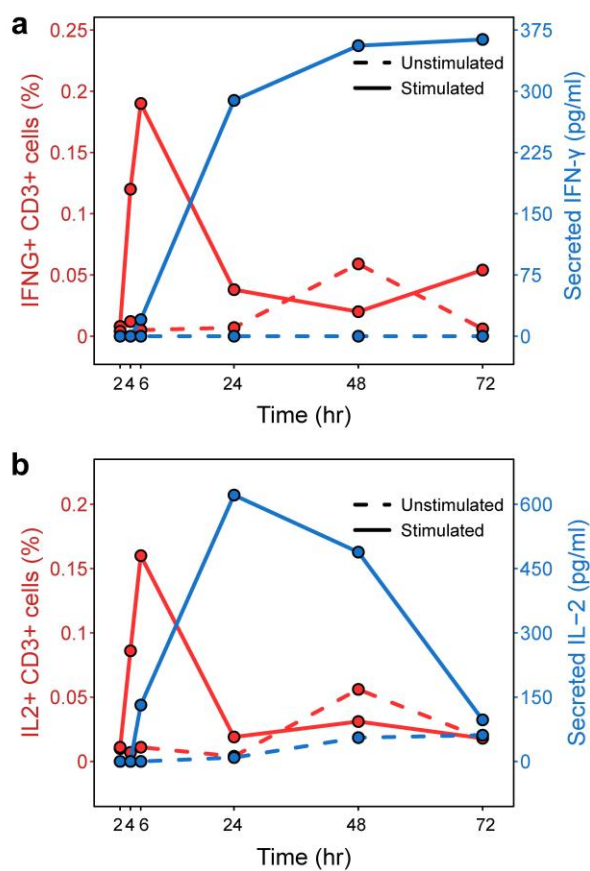

**S2 Fig. Time course of cytokine mRNA induction and corresponding protein secretion.**

Supplement: S2 Fig — PBMCs from an LTBI+ donor were stimulated with 10 μg/ml PPD for the indicated times. Stimulated T cells were analyzed for cytokine mRNA by FISH-Flow and cell-free culture supernatants from the same wells were assayed for cytokine protein levels by ELISA. (a) IFN-γ and (b) IL-2. In both panels and for each time point, the right vertical axis (red) shows the frequency of cytokine mRNA expressing CD3+ cells, while the left vertical axis (blue) shows the concentration of secreted protein (in pg/ml). (PDF) [file pone.0144904.s002.pdf]

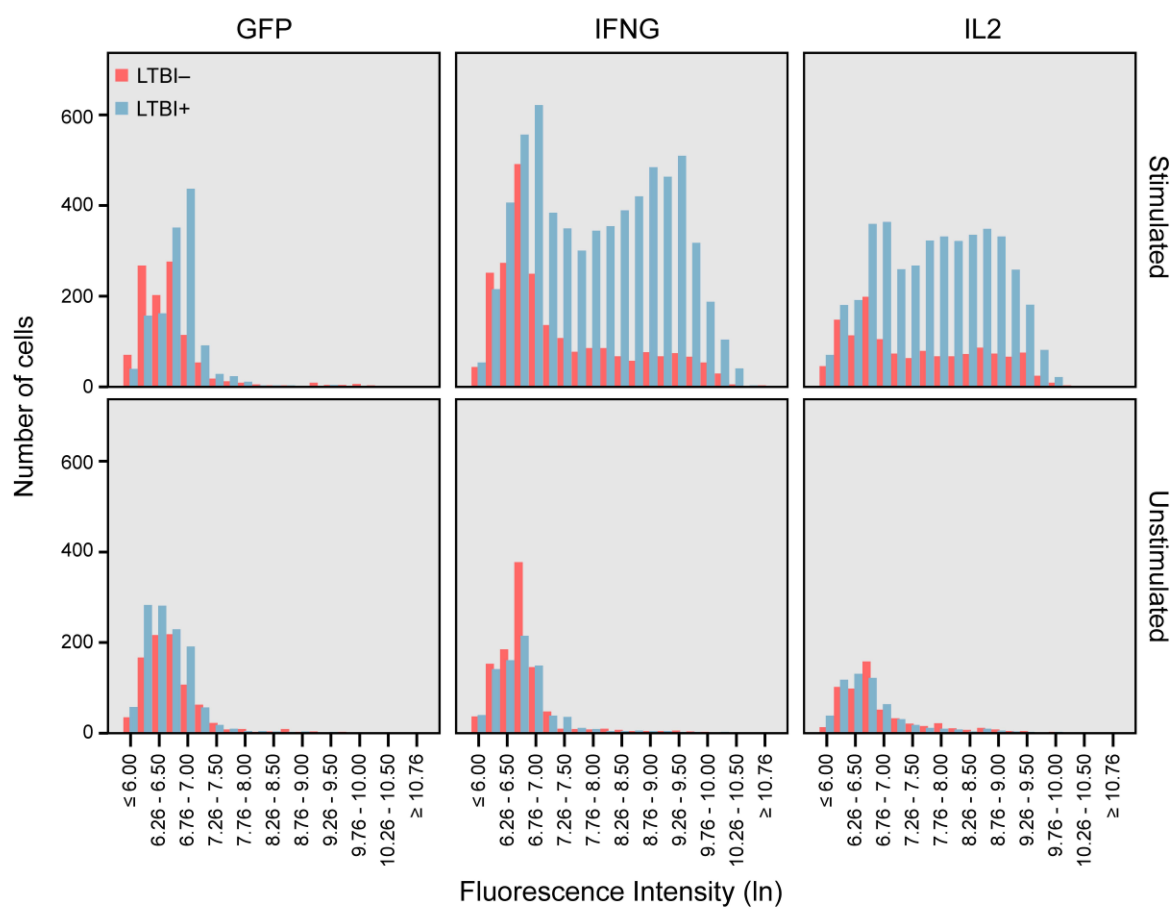

**S3 Fig. Cy5 fluorescence intensity of mRNA+CD3+ cells in LTBI+ and LTBI- donors.**

Supplement: S3 Fig — PBMCs from 33 LTBI+ and 32 LTBI- donors were cultured +/- PPD, stained with FITC-labeled αCD3 antibody, hybridized with Cy5-labeled nucleic acid probes specific for GFP, IFNG and IL2, and analyzed by flow cytometry, as detailed in the legend to Fig 1. The Cy5 fluorescence intensity value of each mRNA+CD3+ cell was extracted using FlowJo software. The graphs show histograms of log-transformed fluorescence data for each gene, unstimulated and PPD stimulated, from LTBI+ and LTBI- donors. Each bin of the histograms comprises interval values of 0.25. (PDF) [file pone.0144904.s003.pdf]

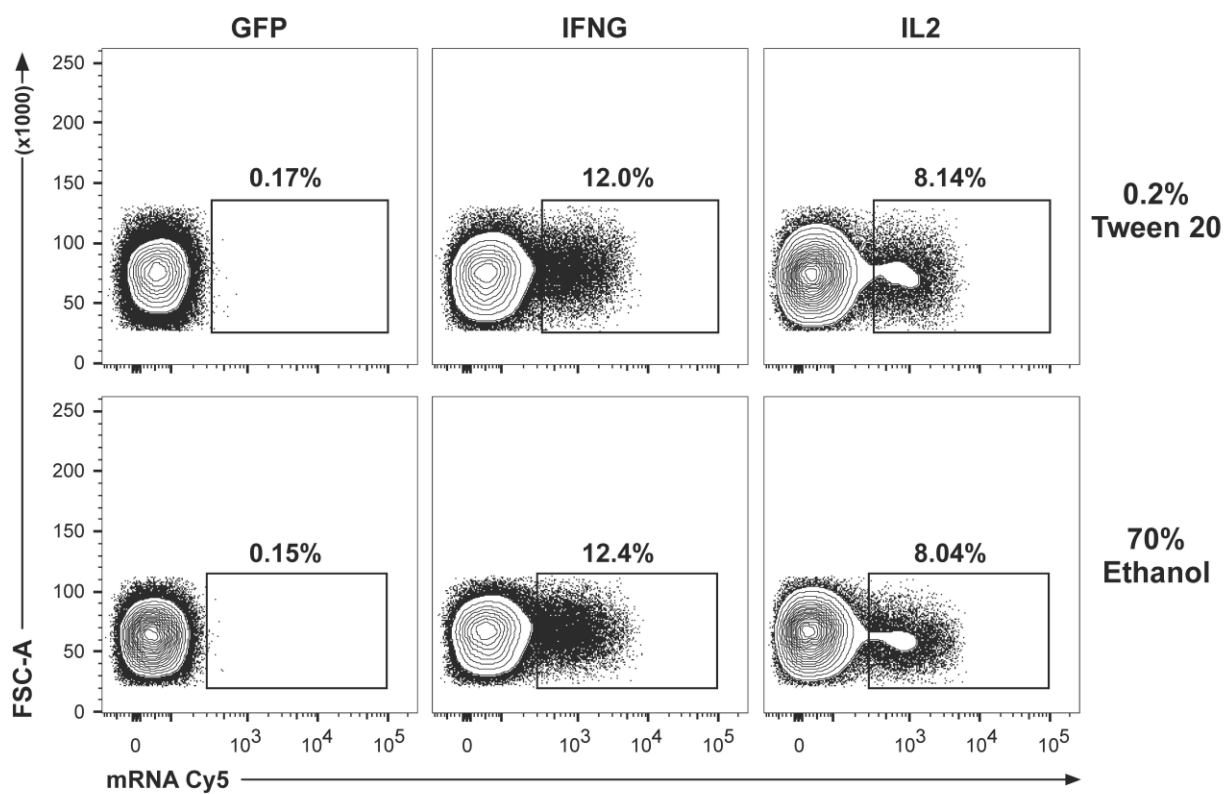

**S4 Fig. Comparison of permeabilization buffers containing 70% ethanol or 0.2% Tween 20.**

Supplement: S4 Fig — PBMCs were stimulated for 2 hr with PMA and Ionomycin, fixed in 4% PFA, and permeabilized for 30 min at room temperature with 0.2% Tween 20 (top row) or 70% ethanol (bottom row). After washes, cells were hybridized with Cy5-labeled GFP, IFNG, or IL2 nucleic acid probes, and analyzed by flow cytometry. Cells were gated according to the forward and side light scatter characteristics of viable lymphocytes. Gates were set on the basis of the GFP control probe and unstimulated control samples. Frequencies of cells expressing GFP, IFNG and IL2 mRNA are reported above each gate. Data from a representative experiment are shown. Similar results were obtained with PPD-stimulated PBMC from an LTBI+ donor (data not shown). (PDF) [file pone.0144904.s004.pdf]

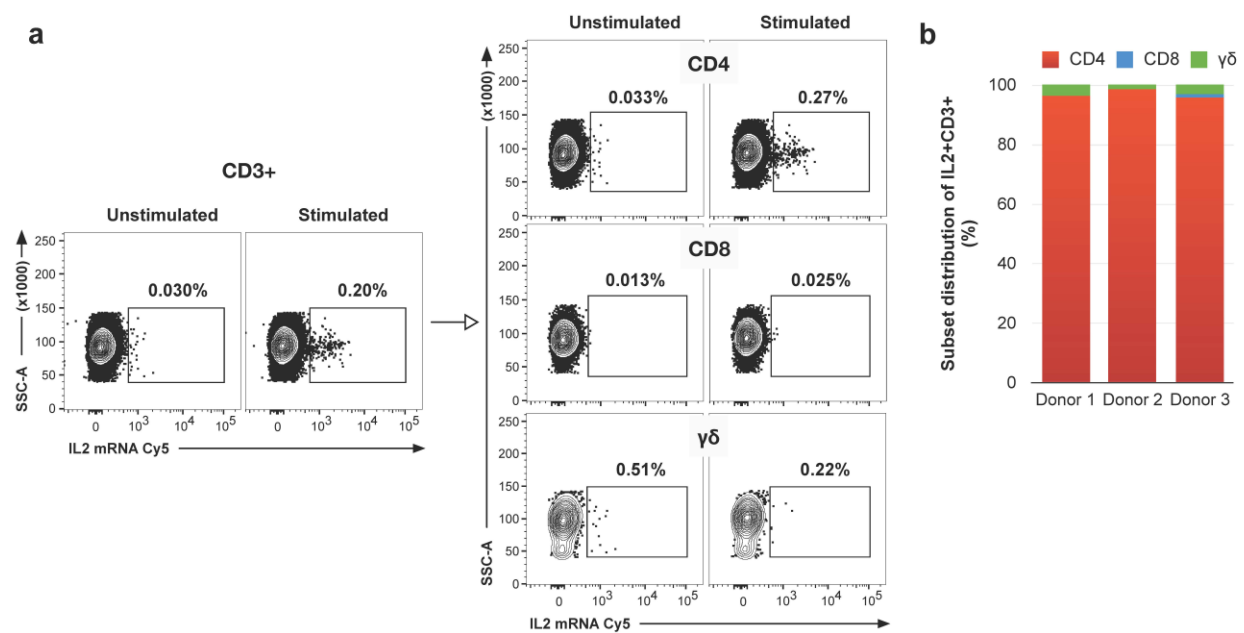

**S5 Fig. Analysis of IL2 expression in CD3+ subsets.**

Supplement: S5 Fig — PBMC from LTBI+ donors were stimulated with PPD or left unstimulated, stained with antibodies against surface markers as indicated, probed with Cy5-labeled IFNG probes, and analyzed by flow cytometry. Gates were set based on unstimulated samples and Fluorescence Minus One (FMO) controls. The scatter plots show data from one donor and the bar graphs from three donors. In each panel, frequencies were calculated relative to the total number of cells in the panel. (a) Frequencies of IL2+CD3+ cells (left panel) and IL2+ cells in the CD4, CD8 and γδ T cell subsets (right panel). (b) Frequency of CD4, CD8, and γδ subsets in IL2+CD3+ cells. (PDF) [file pone.0144904.s005.pdf]

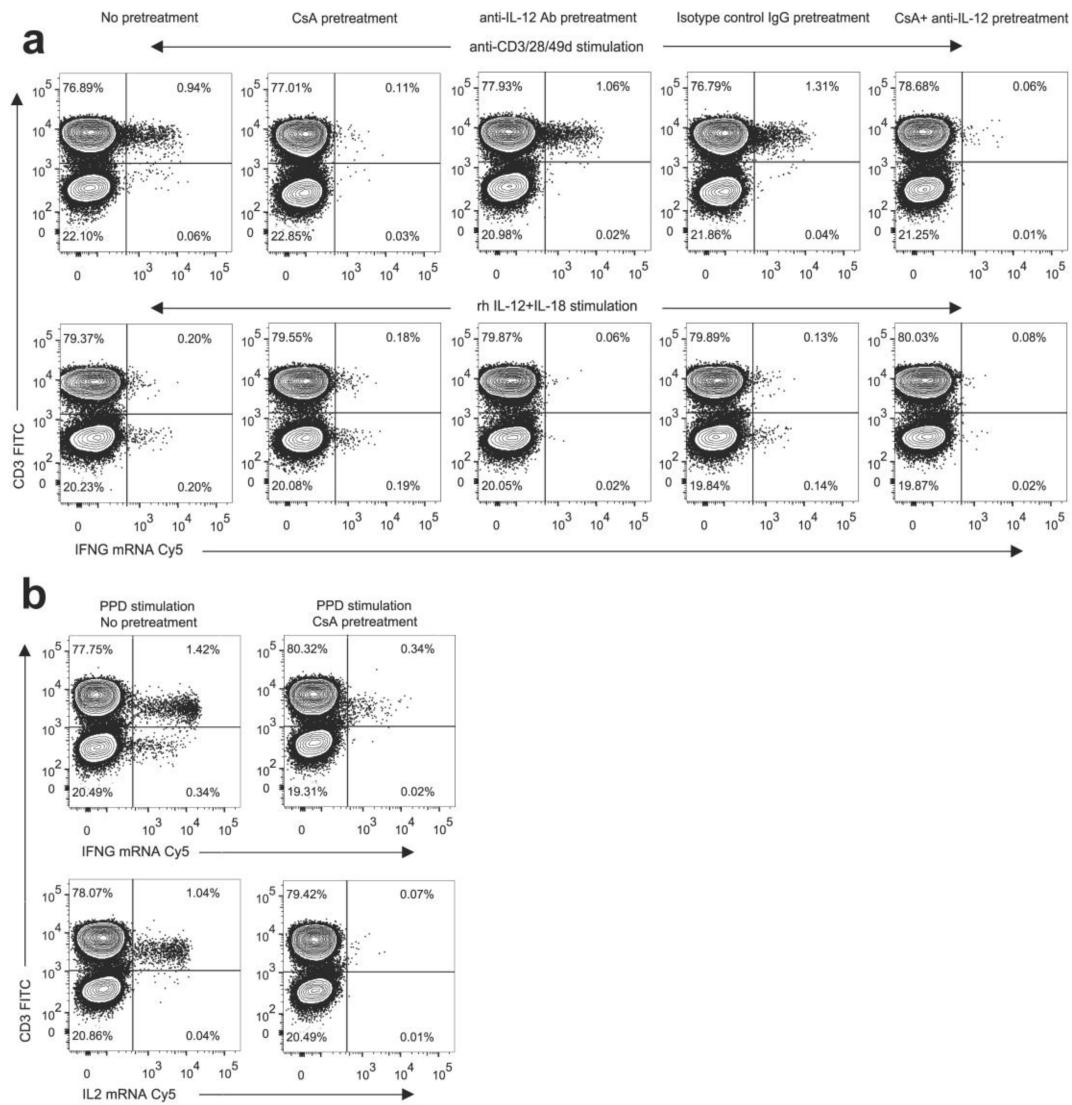

**S6 Fig. Analysis of the mechanism of PPD-induced IFNG expression in T cells.**

Supplement: S6 Fig — (a) PBMCs were stimulated in vitro for 4 hr with either immobilized αCD3 antibody and αCD28/CD49d costimulatory molecules (top row) or with recombinant human IL-12 and IL-18 cytokines (bottom row). Prior to stimulation, cells were subjected to 1-hr treatment at 37°C with CsA, αIL-12 antibody, isotype control antibody, or CsA and αIL-12 antibody together. Gates were established based on unstimulated samples stained with FITC αCD3 antibody and Cy5-labeled IFNG nucleic acid probe. The frequency of IFNG+CD3+ cells is shown in the upper right quadrant of each bichromatic contour plot. (b) PBMCs from an LTBI+ donor were treated with CsA for 1 hr or left untreated, prior to 6 hr PPD stimulation. Stimulated cells were stained with FITC αCD3 antibody, probed with Cy5-labeled nucleic acid probes for IFNG (top panels) or IL2 (bottom panels), and analyzed by flow cytometry. (PDF) [file pone.0144904.s006.pdf]

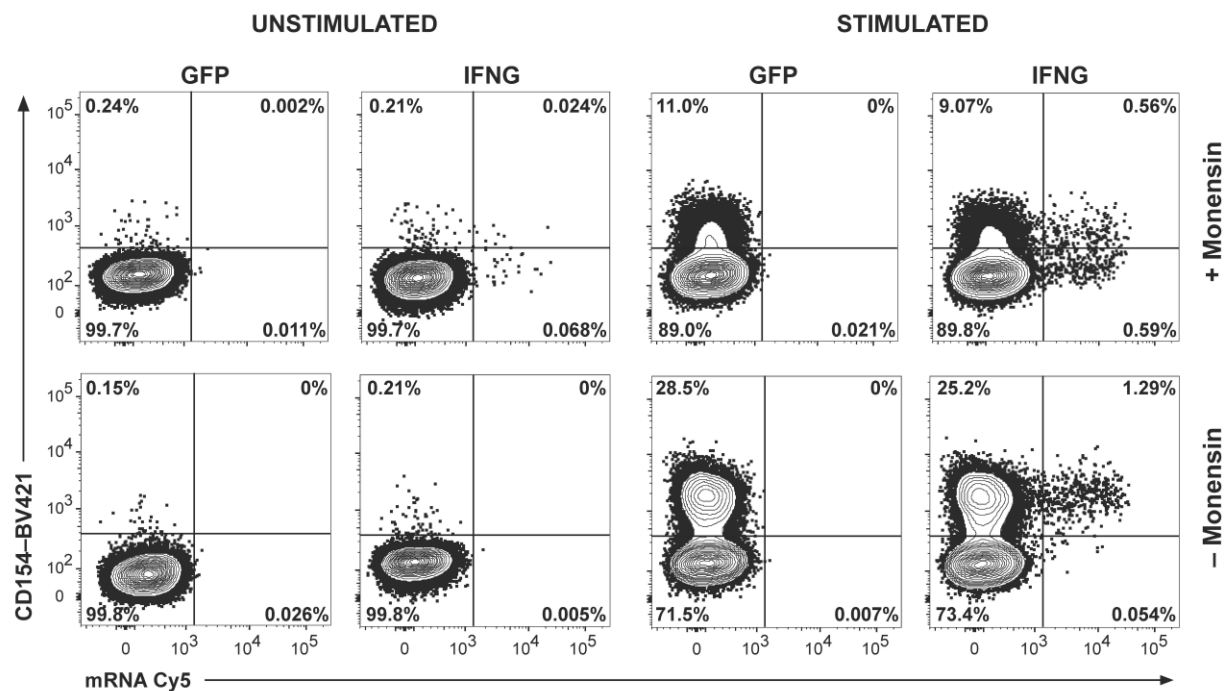

**Fig S7. Surface expression of CD154 activation marker in the presence or absence of monensin.**

Supplement: S7 Fig — PBMCs were stimulated with SEB for 6hr. αCD154 antibody was added during the stimulation (co-culture method), with or without addition of 2 μM monensin, as indicated. Cells were stained for CD3 and CD4 surface markers, probed with Cy5-labeled GFP and IFNG nucleic acid probes, and analyzed by flow cytometry. Gates were set based on unstimulated samples, GFP control probe, and FMO controls. Frequencies of CD4+ T cells are shown in each quadrant. (PDF) [file pone.0144904.s007.pdf]
