# Supplementary material for: Single-Cell Cytokine Gene Expression in Peripheral Blood Cells Correlates with Latent Tuberculosis Status
Source: PLoS One. 2015 Dec 14;10(12):e0144904. doi: 10.1371/journal.pone.0144904 (PMC4681842; doi:10.1371/journal.pone.0144904)
Supplement: S1 Table — (DOCX) [file pone.0144904.s008.docx]

**Table S1 Demographics of the study population**

| **Donor status** |  | **LTBI+ (n=33)** | **LTBI- (n=32)** |
| --- | --- | --- | --- |
| **Gender** | Male | 15 | 20 |
|  | Female | 18 | 12 |
| **Age (years)** | 18-30 | 5 | 9 |
|  | 30-40 | 7 | 12 |
|  | 40-50 | 14 | 4 |
|  | 50-60 | 4 | 6 |
|  | >60 | 3 | 1 |
| **Race** | Asian | 5 | 9 |
|  | Black | 12 | 8 |
|  | White | 16 | 15 |
| **Place of Birth** | U.S.A. | 4 | 10 |
|  | Central/South America | 17 | 7 |
|  | Africa | 6 | 2 |
|  | Asia | 5 | 7 |
|  | Europe | 1 | 6 |
