# Supplementary material for: Single-Cell Cytokine Gene Expression in Peripheral Blood Cells Correlates with Latent Tuberculosis Status
Source: PLoS One. 2015 Dec 14;10(12):e0144904. doi: 10.1371/journal.pone.0144904 (PMC4681842; doi:10.1371/journal.pone.0144904)
Supplement: S2 Table — (DOCX) [file pone.0144904.s009.docx]

**Table S2 Reproducibility of the FISH-Flow assay**

| **Donors** | **Probes** | **IFNG** | | **IL2** | |
| --- | --- | --- | --- | --- | --- |
|  | **Stimulation** | **- PPD** | **+ PPD** | **- PPD** | **+ PPD** |
| **LTBI+ 1** | 1st Donation | 0.02 | 0.24 | 0.02 | 0.13 |
|  | 2nd Donation | 0.04 | 0.86 | 0.01 | 0.45 |
| **LTBI+ 2** | 1st Donation | 0.01 | 0.45 | 0.04 | 0.11 |
|  | 2nd Donation | 0.03 | 0.51 | 0.04 | 0.36 |
| **LTBI+ 3** | 1st Donation | 0.01 | 0.48 | 0.02 | 0.24 |
|  | 2nd Donation | 0.04 | 0.22 | 0.03 | 0.18 |
| **LTBI- 1** | 1st Donation | 0.04 | 0.04 | 0.02 | 0.02 |
|  | 2nd Donation | 0.03 | 0.01 | 0.01 | 0.01 |
| **LTBI- 2** | 1st Donation | 0.02 | 0.02 | 0 | 0.01 |
|  | 2nd Donation | 0.05 | 0.01 | 0.07 | 0.04 |

Values indicate the frequency of cytokine mRNA+ cells from three LTBI+ and two LTBI- donors, obtained from two blood samples per donor drawn >4 months apart.
